# Supplementary material for: Upscaling the Surveillance of Tick-Borne Pathogens in the French Caribbean Islands
Source: Pathogens. 2020 Mar 1;9(3):176. doi: 10.3390/pathogens9030176 (PMC7157729; doi:10.3390/pathogens9030176)
Supplement: Supplementary file 1 [file pathogens-09-00176-s001.pdf]

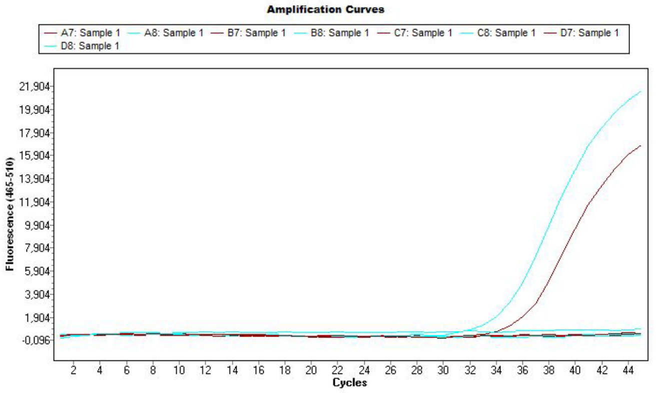

(a)

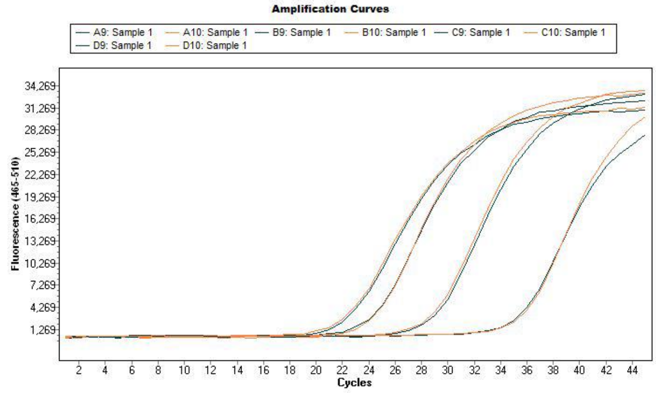

(b)

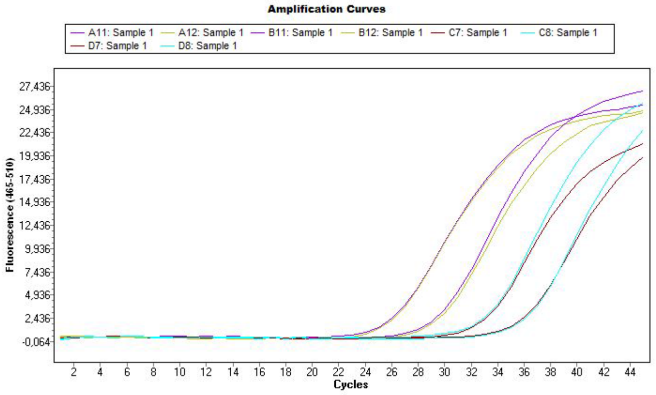

(c)

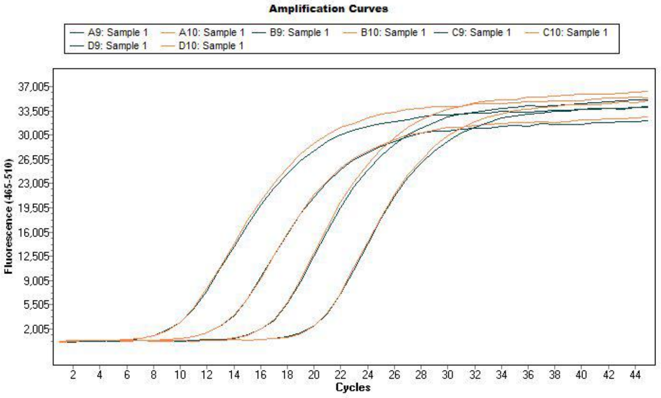

(d)

Figure S1. Improvement of detection signals by pre-amplification. Test of primer/probe set sensitivity for a range of dilutions of positive controls by TaqMan real-time PCR using LightCycler 480, before and after pre-amplification. Results of the sensitivity test of the *Leishmania infantum* design using *a Leishmania infantum* culture, before (a) and after (c) pre-amplification; Results of the sensitivity test of the *Rickettsia* spp. design using *Rickettsia conorii*-positive controls (extracted from an infected *Rhipicephalus sanguineus* tick), before (b) and after (d) pre-amplification.

Table S1. List of the positive control samples used for the Biomark system development.

Positive control	Sample type	Source
Borrelia burgdorferi s.t.	Culture (Strain B31)	National Reference Center of Borrelia (Strasbourg, France)
Borrelia garinii	Culture (Strain N11)	Lise Gern, University of Neuchâtel (Neuchâtel, Switzerland)
Borrelia afzelii	Culture (Strain NE632)	Lise Gern, University of Neuchâtel (Neuchâtel, Switzerland)
Borrelia lusitaniae	Culture (Strain Poti-B1)	Lise Gern, University of Neuchâtel (Neuchâtel, Switzerland)
Borrelia lonestari	Infected *Amblyomma americanum* (Tick collected in USA)	CDC (Atlanta, USA)
Borrelia bissettii	Plasmid, rpoB gene & pBluescriptIISK+	GeneCust, September 2012 (Paris, France)
Borrelia anserina	Plasmid, fla gene & pBluescriptIISK+	GeneCust, December 2015 (Paris, France)
Borrelia parkeri	Culture	Cecilia Hizo-Teufel, National Reference Center of Borrelia (Germany)
Borrelia recurrentis	Culture	ANSES (Maisons-Alfort, France)
Anaplasma marginale	Experimentally infected cow blood	Isabel Garcia Fernandez de Mera (Spain)
Anaplasma phagocytophilum	Infected *Ixodes* spp. (Tick collected in USA)	CDC (Atlanta, USA)
Anaplasma platys	Infected dog blood	H.J. Boulouis, ANSES (Maisons-Alfort, France)
Anaplasma ovis	Plasmid, msp4 gene & pBluescriptIISK+	GeneCust, September 2012 (Paris, France)
Anaplasma bovis	Plasmid, groEL gene & pBluescriptIISK+	GeneCust, January 2016 (Paris, France)
Ehrlichia ewingii	Infected *Amblyomma americanum* (Tick collected in USA)	CDC (Atlanta, USA)
Ehrlichia chaffensis	Infected *Amblyomma americanum* (Tick collected in USA)	CDC (Atlanta, USA)
Ehrlichia ruminantium	Culture	CIRAD (Guadeloupe, France)
Panola Mountain *Ehrlichia*	Infected *Amblyomma americanum* (Tick collected in USA)	CDC (Atlanta, USA)
Ehrlichia canis	Culture	ANSES (Maisons-Alfort, France)
Neoehrlichia mikurensis	Infected rodent blood	Lars, Raberg, University of Lund (Sweden)
Rickettsia conorii	Infected *Rhipicephalus sanguineus* (Tick collected in USA)	CDC (Atlanta, USA)
Rickettsia slovaca	Culture	National Reference Center of Rickettsia (Marseille, France)
Rickettsia massiliae	Culture	National Reference Center of Rickettsia (Marseille, France)
Rickettsia ricketsii	Plasmid, ITS & pBluescriptIISK+	GeneCust, Janvier 2016 (Paris, France)
Rickettsia africae	Culture	National Reference Center of Rickettsia (Marseille, France)
Rickettsia typhi	Culture	ANSES (Maisons-Alfort, France)
Rickettsia prowazekii	Plasmid, gltA gene & pBluescriptIISK+	GeneCust, December 2016 (Paris, France)
Rickettsia felis	Culture	National Reference Center of Rickettsia (Marseille, France)
Bartonella henselae	Culture	ANSES (Maisons-Alfort, France)
Bartonella quintana	Culture	H.J. Boulouis, ANSES (Maisons-Alfort, France)
Bartonella bacilliformis	Culture	ANSES (Maisons-Alfort, France)
Bartonella vinsonii subsp *berkhoffii*	Culture	Lynn Osikowicz, stacey Bartlett, CDC (Colorado, USA)
Francisella tularensis	Culture	Nora Madani, ANSES (Maisons-Alfort, France)
Coxiella burnetti	Culture	Elodie Rousset, ANSES (Maisons-Alfort, France)
Aegyptianella pullorum	Plasmid, groEL gene & pBluescriptIISK+	GeneCust, December 2015 (Paris, France)
Babesia divergens	Culture (clone RFS)	Laurence Malandrin, Oniris (Nantes, France)
Babesia microti	Culture (isolate R1)	Emmanuel Cornillot, University of Montpellier (Montpellier, France)
Babesia bovis	Plasmid, CCTeta gene & pBluescriptIISK+	GeneCust, Janvier 2016 (Paris, France)
Babesia canis	Infected dog blood	VetAgroSup (Lyon, France)
Babesia vogeli	Infected dog blood	VetAgroSup (Lyon, France)
Babesia Rossi	Infected dog blood	ANSES (Maisons-Alfort, France)
Babesia caballi	Plasmid, Rap1 gene & pBluescriptIISK+	GeneCust, September 2012 (Paris, France)
Babesia bigemina	Plasmid, 18S rRNA gene & pBluescriptIISK+	GeneCust, September 2012 (Paris, France)
Babesia ovis	Plasmid, 18S rRNA gene & pBluescriptIISK+	GeneCust, September 2012 (Paris, France)
Babesia duncani	Plasmid, ITS2 & pBluescriptIISK+	GeneCust, January 2016 (Paris, France)
Babesia gibsoni	Plasmid, rap gene & pBluescriptIISK+	GeneCust, December 2015 (Paris, France)
Theileria parva	Culture	Dirk Dobbelaera, University of Bern (Bern, Switzerland)
Theileria annulata	Culture	Dirk Dobbelaera, University of Bern (Bern, Switzerland)
Theileria Lestoquari	Culture	Dirk Dobbelaera, University of Bern (Bern, Switzerland)
Cytauxzoon felis	Plasmid, 18S rRNA gene & pBluescriptIISK+	GeneCust, December 2015 (Paris, France)
Hepatozoon canis	Infected dog blood	Domenico Otranto, Alessio Giannelli, University of Bari (Bari, Italia)
Leishmania infantum (chagasi)	Culture	KASBARI Mohamed, ENVA (Maisons-Alfort, France)
Leishmania Martiniquensis	Culture	KASBARI Mohamed, ENVA (Maisons-Alfort, France)
Rangelia vitalii	Plasmid, 18S rRNA gene & pBluescriptIISK+	GeneCust, December 2015 (Paris, France)

Borrelia theileri	Plasmid, glpQ gene & pBluescriptIISK+	GeneCust, December 2017 (Paris, France)
Theileria mutans	Plasmid, ITS & pBluescriptIISK+	GeneCust, December 2017 (Paris, France)
Theileria velifera	Plasmid, 18S rRNA gene & pBluescriptIISK+	GeneCust, December 2017 (Paris, France)
Theileria equi	Plasmid, ema1 gene & pBluescriptIISK+	GeneCust, December 2017 (Paris, France)
Hepatozoon americanum	Plasmid, 18S rRNA gene & pBluescriptIISK+	GeneCust, December 2017 (Paris, France)
Amblyomma variegatum	Tick DNA (Tick collected in Guadeloupe)	Emmanuel Albina, CIRAD (Guadeloupe, France)
Rhipicephalus microplus	Tick DNA (Tick collected in Gualapagos)	ANSES (Maisons-Alfort, France)
Rhipicephalus sanguineus s.l.	Tick DNA (Tick collected in France)	ANSES (Maisons-Alfort, France)

Table S2. GPS coordinates of the tick collection sites and number of ticks collected. A total of 578 adult ticks collected from cattle from Guadeloupe and Martinique were used for the screening of tick-borne pathogens with the newly implemented BioMark™ real-time PCR system.

Location	Collection Sites	Cattle	Animal	Tick species	Tick number
GUADELOUPE					297
				Amblyomma variegatum	132
				Rhipicephalus microplus	165
	Sainte-Anne (N 16° 14'; W 61° 23')				42
		Cattle 1	Animal 1	*Amblyomma variegatum*	19
		Cattle 2	Animal 2	*Amblyomma variegatum*	2
				Rhipicephalus microplus	4
			Animal 3	*Amblyomma variegatum*	7
				Rhipicephalus microplus	9
		Cattle 3	Animal 4	*Amblyomma variegatum*	1
	Petit Bourg (N 16° 10'; W 61° 36')				48
		Cattle 4	Animal 5	*Amblyomma variegatum*	11
			Animal 6	*Amblyomma variegatum*	9
				Rhipicephalus microplus	5
		Cattle 5	Animal 7	*Amblyomma variegatum*	4
				Rhipicephalus microplus	1
			Animal 8	*Amblyomma variegatum*	13
		Cattle 6	Animal 9	*Amblyomma variegatum*	1
			Animal 10	*Amblyomma variegatum*	4
	Capesterre (N 16° 2'; W 61° 34')				50
		Cattle 7	Animal 11	*Amblyomma variegatum*	9
			Animal 12	*Amblyomma variegatum*	1
				Rhipicephalus microplus	2
		Cattle 8	Animal 13	*Amblyomma variegatum*	1
				Rhipicephalus microplus	16
			Animal 14	*Amblyomma variegatum*	6
				Rhipicephalus microplus	1
		Cattle 9	Animal 15	*Amblyomma variegatum*	2
				Rhipicephalus microplus	5
			Animal 16	*Rhipicephalus microplus*	7
	Le Gosier (N 16° 13'; W 61° 28')				27
		Cattle 10	Animal 17	*Amblyomma variegatum*	11
				Rhipicephalus microplus	3
			Animal 18	*Amblyomma variegatum*	7
		Cattle 11	Animal 19	*Amblyomma variegatum*	1
			Animal 20	*Amblyomma variegatum*	3
		Cattle 12	Animal 21	*Amblyomma variegatum*	2
	Pointe-Noire (N 16° 13'; W 61° 45')				13
		Cattle 13	Animal 22	*Amblyomma variegatum*	2
			Animal 23	*Amblyomma variegatum*	2
			Animal 24	*Amblyomma variegatum*	4
			Animal 25	*Amblyomma variegatum*	2
		Cattle 14	Animal 26	*Amblyomma variegatum*	2
			Animal 27	*Amblyomma variegatum*	1
	Les Abymes (N 16° 16'; W 61° 31')				68
		Cattle 15	Animal 28	*Amblyomma variegatum*	1
		Cattle 16	Animal 29	*Rhipicephalus microplus*	10
			Animal 30	*Rhipicephalus microplus*	6
		Cattle 17	Animal 31	*Rhipicephalus microplus*	18
			Animal 32	*Rhipicephalus microplus*	31
		Cattle 18	Animal 33	*Rhipicephalus microplus*	2
	Morne-À-l'Eau (N 16° 19'; W 61° 28')				46

		Cattle 19	Animal 34	*Rhipicephalus microplus*	6
			Animal 35	*Rhipicephalus microplus*	2
		Cattle 20	Animal 36	*Rhipicephalus microplus*	19
			Animal 37	*Rhipicephalus microplus*	7
		Cattle 21	Animal 38	*Amblyomma variegatum*	1
				Rhipicephalus microplus	11
	Bouillante (N 16° 7'; W 61° 46')				3
		Cattle 22	Animal 39	*Amblyomma variegatum*	2
			Animal 40	*Amblyomma variegatum*	1
MARTINIQUE					281
				Rhipicephalus microplus	281
	Rivière-Pilote (N 14° 28', W 60° 54')				4
		Cattle 1	Animal 1	*Rhipicephalus microplus*	4
	Le Diamant (N 14° 29'; W 61° 1')				1
		Cattle 2	Animal 2	*Rhipicephalus microplus*	1
	Le François (N 14° 36'; W 60° 53')				52
		Cattle 3	Animal 3	*Rhipicephalus microplus*	50
		Cattle 4	Animal 4	*Rhipicephalus microplus*	2
	Le Lamentin (N 14° 37'; W 60° 59')				2
		Cattle 5	Animal 5	*Rhipicephalus microplus*	2
	Le Lorrain (N 14° 49'; W 61° 2')				5
		Cattle 6	Animal 6	*Rhipicephalus microplus*	5
	Le Morne-Vert (N 14° 42'; W 61° 8')				3
		Cattle 7	Animal 7	*Rhipicephalus microplus*	3
	Le Robert (N 14° 40'; W 60° 56')				57
		Cattle 8	Animal 8	*Rhipicephalus microplus*	47
		Cattle 9	Animal 9	*Rhipicephalus microplus*	3
		Cattle 10	Animal 10	*Rhipicephalus microplus*	6
		Cattle 11	Animal 11	*Rhipicephalus microplus*	1
	Le Vauclin (N 14° 32'; W 60° 50')				40
		Cattle 12	Animal 12	*Rhipicephalus microplus*	12
		Cattle 13	Animal 13	*Rhipicephalus microplus*	6
		Cattle 14	Animal 14	*Rhipicephalus microplus*	3
		Cattle 15	Animal 15	*Rhipicephalus microplus*	4
		Cattle 16	Animal 16	*Rhipicephalus microplus*	2
		Cattle 17	Animal 17	*Rhipicephalus microplus*	5
		Cattle 18	Animal 18	*Rhipicephalus microplus*	3
		Cattle 19	Animal 19	*Rhipicephalus microplus*	5
	Rivière-Salée (N 14° 31'; W 60° 57')				3
		Cattle 20	Animal 20	*Rhipicephalus microplus*	3
	Saint-Esprit (N 14° 33'; W 60° 55')				28
		Cattle 21	Animal 21	*Rhipicephalus microplus*	28
	Saint-Joseph (N 14° 40'; W 61° 2')				13
		Cattle 22	Animal 22	*Rhipicephalus microplus*	7
		Cattle 23	Animal 23	*Rhipicephalus microplus*	6
	Sainte-Anne (N 14° 26'; W 60° 50')				29
		Cattle 24	Animal 24	*Rhipicephalus microplus*	3
		Cattle 25	Animal 25	*Rhipicephalus microplus*	8
		Cattle 26	Animal 26	*Rhipicephalus microplus*	18
	Sainte-Luce (N 14° 29'; W 60° 56')				36
		Cattle 27	Animal 27	*Rhipicephalus microplus*	3
		Cattle 28	Animal 28	*Rhipicephalus microplus*	33
	Sainte-Marie (N 14° 46'; W 60° 59')				8
		Cattle 29	Animal 29	*Rhipicephalus microplus*	8
Total					578
